# Canonical Correlation between Behavioral-Psychological Variables and Predictors of Coronary Artery Disease Prognosis

**DOI:** 10.3390/ijerph17051608

**Published:** 2020-03-02

**Authors:** Chul-Hoon Kim, In-Kyoung Noh, Jung Mi Ryu, Eun Jung Bae, Hoo Jeung Cho, Myoung Soo Kim

**Affiliations:** 1 College of Medicine, Dong-A University, Busan 49201, Korea; bbp2000@hanmail.net; 2 Department of Internal Medicine, Kosin University Gospel Hospital, Busan 49267, Korea; ada10kr@naver.com; 3 Department of Nursing, Pukyong National University, Busan 48513, Korea; rewmis@naver.com (J.M.R.); ccu0401@naver.com (H.J.C.); 4 Department of Nursing, Dongnam Institute of Radiological & Medical Sciences, Busan 46033, Korea; beaulife-@hanmail.net

**Keywords:** coronary artery disease, lifestyle, metabolic syndrome, severity of illness index, type-D personality

## Abstract

Metabolic syndrome (MetS) and severity of coronary artery disease (CAD) are considered predictors of CAD prognosis. Unhealthy lifestyles and type-D personality are associated with MetS and are potential causes of primary and secondary CAD. In this cross-sectional descriptive study, we aimed to investigate the relationship between behavioral-psychological variables and predictors of CAD prognosis. The behavioral-psychological variable set contained six lifestyle categories and two type-D personality categories. Descriptive analyses, t-tests, analysis of variance, Pearson’s correlation, and canonical correlation were used. The behavioral-psychological variable set was related to the predictor set for CAD prognosis, with a significant canonical variate of 0.67 (45% overlapping variance). Significant pairs of canonical variates indicated that poor physical activity and weight control (−0.77), poor dietary habits (−0.78), alcohol consumption and cigarette smoking (−0.37), lack of sleep and rest (−0.40), stress (−0.64) in the lifestyle set, higher negative affectivity (0.52), and social inhibition (0.71) in the type-D personality set were associated with a high MetS score (0.59) and severity of CAD (0.91). A combination of behavioral and psychological variables was found to be important in predicting the prognosis of CAD; therefore, interventions aimed at preventing combinations of these variables may be effective in improving CAD prognosis.

## 1. Introduction

Coronary artery disease (CAD) is the most common type of ischemic heart disease. This progressive and recurrent disease may present with atherosclerotic or non-atherosclerotic coronary arteries [1]. Ischemic heart disease occurs not only owing to sclerosis of the coronary artery but also due to functional coronary vasomotion, including vascular tone and coronary artery spasm [2]. Therefore, the management of CAD should involve improving the prognosis of patients with CAD by modifying other detrimental factors and addressing issues arising within the diseased coronary artery. Healthcare costs related to morbidity and mortality owing to CAD reportedly increase social and economic burdens [3]. However, the mortality rate 2–3 years following CAD onset has been found to be similar to that of the general population [4]; therefore, healthcare providers need be able to provide patients with CAD and their families with appropriate prognostic information. One prognostic predictor of CAD is metabolic syndrome (MetS). MetS components can create a state of oxidative stress that is linked to ischemic heart disease [5,6] and that increases a patient’s risk for primary CAD [7]. Furthermore, the presence of MetS in young patients with CAD has been found to be an important predictor of six-year major cardiac events (hazard ratio 3.32) and repeated myocardial infarction (hazard ratio 7.78) [8]. Angiographic severity of CAD is another predicting factor, which is indicative of the recurrent development of coronary atherosclerosis [9]. The severity of physiological stenosis has also been shown to have a significant association with the risk of clinical events and provide better prognostic stratification [10]. 

Unhealthy behaviors have been associated with the prognosis of CAD [11]. Guidelines recommend 30 min of moderate-intensity physical activity five days a week; however, non-adherence to exercise remains problematic [12] despite physical activity playing a critical role in the secondary prevention of cardiovascular diseases [13]. Sedentary lifestyles, such as sitting for a total of 6–7 h/day and 3–4 h/day of TV viewing, have been associated with increased cerebrovascular disease-related mortality [14]. Moreover, based on angiography reports, the most prevalent type of coronary obstruction is due to abnormal lipid profiles [15], which are often related to unhealthy lifestyle behaviors such as cigarette smoking and an increased consumption of fat and sugar [16]. In addition, both short and long sleep durations have been independently associated with higher all-cause mortality [17]; therefore, behavior modification needs to be implemented to help prevent poor prognoses for patients with CAD.

According to a systematic review concerning risk factors for CAD, individual psychological factors have also been found to be associated with prognosis and health outcomes, including re-hospitalization and mortality [18]. Psychological factors such as depression, anxiety, social isolation, psychosocial distress, and a type-D personality [18], which are known risk factors for cardiovascular disease [19], have been reported to increase the risk of developing CAD by 32.5% [20]. Depression, distress, and anxiety are factors that have been reported to contribute to heart failure [21] and atrial fibrillation [6] through several mechanisms. Individuals with a type-D personality are defined as those with a tendency toward higher stress and anger levels, often because of psychological depression [22]. With respect to a type-D personality, psychophysiological and behavioral pathways have been identified as two potential mechanisms affecting the development of heart disease [23]. The psychophysiological pathway could involve an elevated cortisol awakening response that was mediated by the hypothalamic–pituitary–adrenal axis in patients with acute coronary syndrome and a type-D personality [24]. The behavioral pathway may contribute to the development of CAD through behavioral issues such as sedentary lifestyle [25] and an unhealthy diet [26]. Specifically, the combined effect of these two pathways may increase the risk of a cardiovascular event, and behavioral issues have been shown to lead to advanced complications of cardiovascular disease later in life [27]. 

Although several studies have demonstrated the role of behavioral-psychological factors on CAD [28,29], empirical studies on predictors of CAD prognosis such as MetS and its relationship with the severity of CAD are lacking, and their results are inconsistent [27,30]. Furthermore, the interconnections of different behavioral-psychological factors with different MetS components and the severity of CAD have not been investigated in detail. Type-D personality traits have been shown to be related to maladaptive health-related behavior [31] and are associated with an increased severity of CAD [13]; therefore, assessing combinations of behavioral and psychological variables could be expected to provide more extensive information than a bivariate relationship with MetS components and CAD severity alone and may help refine guidelines for patients at a risk of CAD. Therefore, two research questions were set: is there a relationship between behavioral-psychological variables and predictors for CAD prognosis? If so, which combination is most strongly related to CAD prognosis? 

## 2. Materials and Methods 

### 2.1. Study Design and Participants

**Figure 1:**
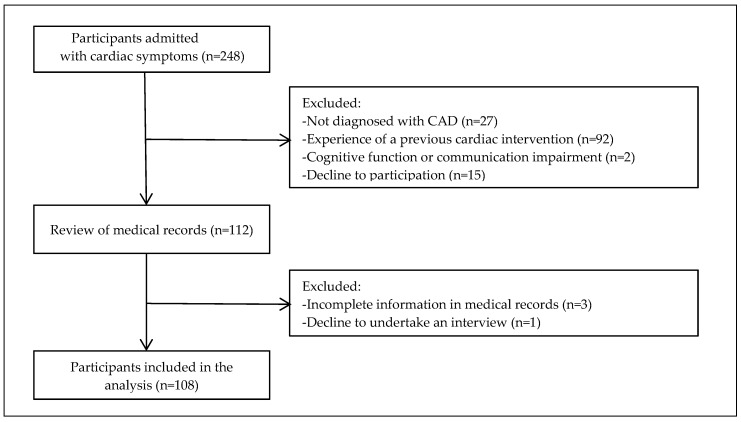
Flowchart of study population.

This cross-sectional descriptive study involved interviews using a structured survey and a review of medical records. Patients were recruited from a cardiology outpatient department at a tertiary hospital in Korea. Inclusion criteria comprised participants diagnosed with CAD [stable angina pectoris, unstable angina pectoris, ST segment -elevation myocardial infarction (STEMI), and non-ST elevation myocardial infarction (NSTEMI)] based on coronary angiography results, those who had not undergone a previous cardiac intervention, those with no cognitive function or communication impairments, and those who consented to participation in this study. Exclusion criteria comprised participants with incomplete information in their medical records and those who declined to undertake an interview. Figure 1 shows the flowchart of the study population. The required sample size in a canonical correlation is 10 times per variable [32]. This study comprised 108 participants, which was sufficient for the analysis of 10 variables (six variables for behavior components, two variables for type-D personality, and two predictive variables for CAD prognosis).

### 2.2. Measurements

#### 2.2.1. Sociodemographic and Disease-Related Characteristics

All participant characteristics were obtained at the time of diagnosis from the survey and the medical record data. Sociodemographic characteristics included the following six items: age, sex, educational status, marital status, subjective economic status, and health status. Subjective economic status and health status were classified as low (coded 1), moderate (coded 2), and high (coded 3). Disease-related characteristics included the following six items: cigarette smoking history, family history, type of CAD, and the use of antihypertensive, antidiabetic, and antihyperlipidemic medication. Cigarette smoking history was classified as follows: non-smoker, past smoker, and current smoker. Family history was assessed to determine whether there was a family history of CAD. The type of CAD was classified into two categories, namely angina pectoris, including stable angina pectoris, and acute coronary syndrome, including unstable angina pectoris, STEMI, and NSTEMI. The use of medication was classified as either “yes” (coded 1) or “no” (coded 0).

#### 2.2.2. Behavioral-Psychological Variable Set

The behavioral-psychological variable set contained six variables in the lifestyle subcategory and two variables in the type-D personality subcategory. 

##### Lifestyle (Behavioral Set)

A lifestyle evaluation tool for patients with MetS [33] was used to assess participant lifestyle. This 36-item instrument comprised 6 dimensions, namely physical activity and weight control (8 items), dietary habits (16 items), alcohol consumption and cigarette smoking (3 items), sleep and rest (2 items), stress (3 items), and medication and health management (4 items). Item responses were measured using a 4-point scale from 1 (not at all) to 4 (always). Higher scores corresponded to a healthy lifestyle. Internal consistency in the original study reported a value of 0.92 [33], and this study had an internal consistency value of 0.93. 

##### Type-D Personality (Psychological Set) 

We used “The Korean type-D scale-14” that was developed by Denollet [22] and verified in Korean by Lim et al. [34]. This scale comprised two parts, namely negative affectivity and social inhibition, each with 7 items. Assessments were based on a 5-point scale from 0 (definitely disagree) to 4 (definitely agree). Cronbach’s alpha for negative affectivity and social inhibition were 0.88 and 0.86, respectively, in the development study [22], and 0.83 and 0.82, respectively, in this study. 

#### 2.2.3. Predictors of CAD Prognosis Variable Set

The variable set for predictors of CAD prognosis contained two variables, namely the MetS score and the severity of CAD. 

##### MetS Score

To identify MetS, we used the National Cholesterol Education Program-Adult Treatment Panel Ⅲ (NCEP-ATP Ⅲ) [35] for triglycerides, high-density lipoprotein-cholesterol (HDL-C), blood pressure, and fasting glucose. To diagnose MetS, the NCEP-ATP Ⅲ [35] requires the presence at least 3 of the following components: (1) hypertriglyceridemia (triglycerides >150 mg/dL); (2) low HDL-C (HDL-C < 40 mg/dL in men or <50 mg/dL in women); (3) hypertension (systolic blood pressure >130 mmHg or diastolic blood pressure >85 mmHg); (4) hyperglycemia (fasting glucose concentration >100 mg/dL or diagnosed with type 2 diabetes); and (5) abdominal obesity (waist circumference >90 cm in men and >88 cm in women, for Asian populations). Furthermore, treatment with specific medications, including medications used to manage blood pressure, triglycerides, cholesterol, and glucose levels, should be considered. However, due to a lack of waist circumference data recorded over the same period as the other four components, body mass index (BMI) >25 kg/m^2^ was used based on the obesity criterion recommendations of the Korean Society for the Study of Obesity [36]. To calculate the MetS score, each component was classified as yes (coded 1) or no (coded 0). The MetS score ranged from 0 to 5, with a higher score indicating a greater severity MetS.

##### Severity of CAD

To determine the severity of CAD, we used the number of significantly stenotic coronary arteries (including arterial branches), as noted in the medical records. Intravascular ultrasonography was analyzed independently by a radiologist and a physician to characterize the extent and the degree of stenosis. Significant stenosis was defined as > 50% occlusion of the coronary artery’s internal diameter. No significant stenosis vs. significant stenosis was scored as 0 vs. 1; respectively, significant stenosis in 1 vessel vs. 2 vessels vs. 3 vessels was scored as 1 vs. 2 vs. 3, respectively, with the highest score indicating increased severity. 

### 2.3. Ethical Considerations

This study was conducted following a review and approval from the hospital’s Institutional Review Board (IRB no. 1608012044). Prior to data collection, researchers met with the participants and provided them with an information sheet explaining the study aims, the confidentiality of personal information, the anonymity of the survey, and the voluntary nature of participation. Written consent was obtained from participants prior to questionnaire distribution. This study complied ethically with the Declaration of Helsinki.

### 2.4. Data Analysis

Statistical Package for the Social Science (SPSS) version 23.0 (SPSS Inc., Chicago, IL, USA) was used to analyze the data. For descriptive analyses, a t-test or a Mann–Whitney U-test, an analysis of variance or a Kruskal–Wallis test, and a Pearson’s correlation were used. Canonical correlation analysis was performed after the assumptions of the canonical correlation were examined. Pairs of canonical variates were interpreted as reliable if the structure coefficient was >0.30 because an explanatory power > 9% was deemed to be a meaningful value [32].

## 3. Results

### 3.1. Predictors of CAD Prognosis According to Participant Characteristics

Table 1 shows the MetS score and the severity of CAD according to participant characteristics. The mean age of the participants was 62.8 ± 10.5 years, and 51.9% of the participants were male. There were no significant differences in MetS score; however, we observed a significant difference according to subjective health status (*F* = 5.79, *p* = 0.004), antidiabetic medication use (t = −4.01, *p* < 0.001), and antihyperlipidemic medication use (t = −3.18, *p* = 0.002). There were significant differences in the severity of CAD according to age (*H* = 9.66, *p* = 0.022), type of CAD (Z = −2.99, *p* = 0.003), and antihypertensive medication use (Z = −3.39, *p* = 0.001). 

### 3.2. Lifestyle, Type- D Personality, MetS Score, and Severity of CAD

**Figure 2:**
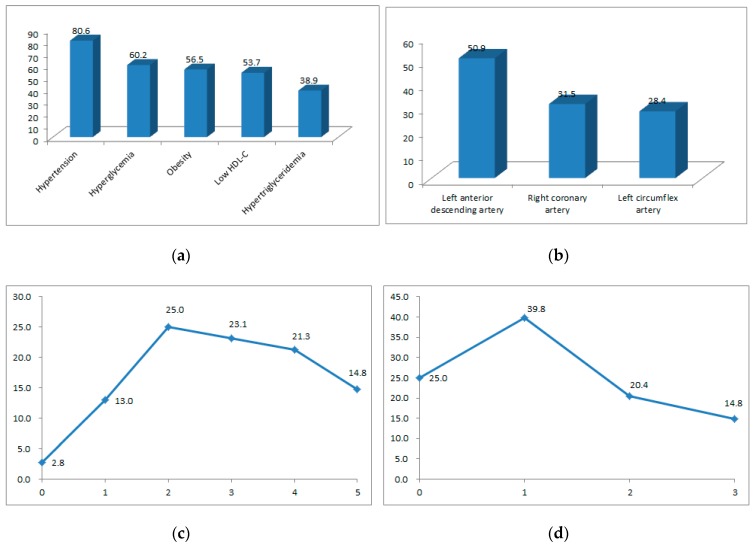
Characteristics of the metabolic syndrome and the severity of coronary artery disease; (**a**) Prevalence of metabolic syndrome components; (**b**) prevalence of coronary artery stenosis; (**c**) proportion of metabolic syndrome score; (**d**) proportion of coronary artery disease severity

The highest lifestyle factor scores were for stress, whereas physical activity and weight control had the lowest lifestyle scores. The mean negative affectivity score was 3.43 ± 0.82, and the mean social inhibition score was 2.71 ± 1.00. The mean MetS score was 2.92 ± 1.35, and the mean severity score for CAD was 1.25 ± 0.99 (Table 2). Figure 2 shows the characteristics of the MetS score and the severity of CAD. For the MetS component assessment, the prevalence rates for hypertension and hypertriglyceridemia were 80.6% and 38.9%, respectively. The proportion of participants with significant stenosis in the left anterior descending artery (LAD) was 50.9%. Approximately 40% of the participants had significant stenosis in at least one vessel, and 14.8% had fixed stenosis in >50% of the internal diameter in three coronary arteries.

### 3.3. Correlation between the Behavioral-Psychological Variable Set and Predictors of CAD Prognosis

Assumption tests were conducted before the canonical correlation analysis. The normality, the linearity, and the homoscedasticity met the assumptions, except for MetS components and the degree of stenosis in each artery. The correlation between behavioral-psychological variables and predictors of CAD prognosis was between 0.20~0.42, which showed an absence of multicollinearity (Table 3).

The behavioral-psychological variable set was related to the predictor set of CAD prognosis with one significant canonical variate of 0.67 (45% overlapping variance) (Table 4). Wilk’s Lambda value with all canonical correlations was 0.51, which was statistically significant (*F* = 4.91, *p* < 0.001). The significant pair of canonical variates indicated that poor physical activity and weight control (−0.77), poor dietary habits (−0.78), alcohol consumption and cigarette smoking (−0.37), lack of sleep and rest (−0.40), stress (−0.64) in the lifestyle set, higher negative affectivity (0.52), and social inhibition (0.71) in the type-D personality set were associated with a high MetS score (0.59) and severity of CAD (0.91). 

## 4. Discussion

Participants who were older, those with acute coronary syndrome, and those with antihypertensive medication use showed a high severity of CAD, however, there was no difference between the sexes in terms of severity of CAD. Compared to age-matched males, premenopausal females have been reported to have a reduced incidence of cardiovascular disease that presented 10 years later than that in men [37] owing to the protective role of estrogen [38], however, a greater number of elderly women were included in this study. Acute coronary syndrome has been reported to have a higher severity of CAD than stable angina. One epidemiological study showed that mortality rates for each acute coronary syndrome (unstable angina pectoris, STEMI, and NSTEMI) were relatively high in the five years after diagnosis (19%, 22%, and 17%, respectively) [39]. Therefore, it is essential that patients with acute coronary syndrome follow stringent risk management to prevent secondary events or death. The use of antihypertensive medication was associated with a higher severity of CAD in this study. Because of the cross-sectional study design, it was not clear whether participants had hypertension and CAD or whether they were participants with hypertension at a high risk for CAD. According to a follow-up study of cardiovascular disease, only 2.1% of the patients had uncontrolled hypertension [40]. A multi-drug blood pressure control strategy that includes angiotensin-converting enzyme inhibition in high-risk patients with CAD has been shown to be beneficial in reducing the risk of CAD [41]. Therefore, more detailed information, including that of the type of medication and dosage, is needed to clarify the relationship between antihypertensive medication and the severity of CAD.

In this study, hypertriglyceridemia was the lowest component of MetS (38.9%), and the mean MetS score was 2.92, which was consistent with trends in the prevalence of MetS as identified in the Korean National Health Insurance Service data [42]. However, the mean triglyceride level was 134.78 mg/dL, which has been defined as a high-normal triglycerides level. In a recent cohort study, there was an elevated risk for death in patients with high-normal triglyceride levels of 100 to 149 mg/dL compared with that in those with lower triglyceride levels [43]. Hypertriglyceridemia had the highest hazard ratios for atheroma progression and the strongest relationship with the prevalence of myocardial infarction and stroke in patients [44]. For patients with cardiovascular disease, regardless of adjustment for HDL-C, elevated triglyceride levels have been associated with an increased risk of all-cause mortality [43]. Triglyceride levels should be managed with greater vigilance. Moreover, accelerated cardiovascular disease progression has been reported to be due to the presence of individual components rather than due to MetS alone [45,46], and care needs to be exercised when using the MetS score with respect to the component management of patients without MetS but who have one or two components.

Participants had significant stenosis in 1.25 vessels on average. Similar to a previous study [15], 39.8% of participants had significant stenosis in a single coronary artery, mostly the LAD. Given that the left ventricle end-diastolic diameter has been found to be inversely related to impaired function in the neuropsychological phase [47], attention should be paid to the severity of LAD stenosis. Endothelial dysfunction induces coronary abnormalities that are critical in cardiovascular stenosis because endothelial cells regulate vascular and inflammatory responses [2]. Specifically, type-D personality is associated with impaired endothelial dysfunction in patients with CAD [48]. The mechanism underlying the influence of type-D personality on endothelial dysfunction remains unclear. However, type-D personality characterized by stress may be considered to indicate a high risk because stress is known to induce endothelial dysfunction [48]. 

Only one pair of canonical variates was significant. The strongest variable combinations were poor physical activity/weight control, unhealthy dietary habits, and social inhibition. A previous study found that people with type-D personalities have less healthy lifestyles involving less physical activity and a less varied diet [31]. A type-D personality is an independent predictor of decreased exercise capacity and decreased motivation for activity [13]. Social inhibition is associated with interpretation biases toward cognitive, affective, and behavioral factors [49], which may make it difficult to motivate patients with depression to modify their behavior. Specifically, due to traits with regard to higher levels of anxiety and depressive mood, social inhibition plays a key role in the higher drop-out rate from cardiac rehabilitation programs [50], and this could be also a potential cause of CAD recurrence. 

Furthermore, type-D personality has been associated with significantly less healthy food intake, including a greater consumption of fat and sugar compared with fruit and vegetable intake [16]. Patients with higher social inhibition have poor dietary habits because they often are non-adherent to recurrence preventive regimens [14]. Saturated fat and sugar can raise total cholesterol levels in the blood, and sugar intake can have unfavorable effects on triglyceride levels [51]. High sugar intake also promotes insulin resistance, which is reported to be one of the main causes of CAD development due to MetS [52]. Therefore, the combination of unhealthy physical activity/dietary habits as behavioral components and social inhibition as the psychological component should be separately identified and addressed to improve the prognosis for patients with CAD. 

A combination of severe stress and negative affectivity has also been found to be related to a poor CAD prognosis. Negative affectivity appears to be a significant component for mental distress [13], and high negative affectivity has been characterized as experiencing enhanced feelings of dysphoria, anxiety, and irritability and a negative feeling towards oneself [22]. Individuals with negative affectivity are likely to be more focused on emotions, and, at the time of stress, cortisol is released as an effector hormone, which influences target organs such as the heart [23], leading to increased inflammation, which plays a critical role in the development of atherosclerosis [53]. As a result, responding emotionally to stressful situations may worsen the CAD prognosis. Therefore, negative affectivity should be controlled so that it does not become associated with higher stress and enhances the likelihood of a poor CAD prognosis. 

Based on our findings, a rehabilitation program that aims to prevent a combination of behavioral and psychological variables is likely to be more effective in improving CAD prognosis and should involve periodic type-D personality screening. In one study, a type-D personality diagnosis was found to have altered almost 60% of patients postoperatively [54]; therefore, appropriate interventions for patients with type-D personalities should be provided. Considering the strong relationship between unhealthy behaviors, social inhibition, and predictors for CAD prognosis, a weaning strategy concerning social inhibition needs to be prioritized for the rehabilitation program. Furthermore, based on the multifaceted model of social inhibition [49], integrative intervention, such as psychodynamic intervention, should be included to reduce cognitive, affective, and behavioral inhibition [55]. Psychodynamic intervention is a supporting strategy for promoting self-management behavior by improving a patient’s cognitive and affective inhibition [55]. Psychodynamic motivation and training programs have shown that improving physical activity after myocardial infarction [56], and a stepwise psychotherapy intervention, including group psychotherapy and incorporating cognitive-behavioral elements, can improve symptoms of depression in patients with CAD [57]. 

## 5. Limitation

This study has several limitations. First, the data were derived from a single cardiology center using a convenience sampling method; therefore, some results of this study may not be generalizable to all Koreans. Thus, future studies should include a greater number of participants and clinics. Second, we could not define the causal relationship between behavioral-psychological change and CAD prognosis because our study was not longitudinal. Therefore, longitudinal screening for behavioral-psychological factors is required when designing future studies. Third, bias may have occurred when evaluating disease-related participant characteristic data extracted from medical records, and recall bias may have occurred when information was obtained from survey responses. Thus, a prospective study design may be merited to eliminate potential bias. Finally, participants’ use of antihyperlipidemic medication was not classified in terms of antitriglyceride and anticholesterol medication because these data were recorded in the outpatient department and not in the medical records. Therefore, participants using antihyperlipidemic medication may have been unnecessarily checked for hypertriglyceridemia and low HDL-C components, and an over-estimation may have been possible when calculating the MetS score. Therefore, these results should be interpreted cautiously.

## 6. Conclusions

Pairs of behavioral-psychological variables, specifically a combination of physical activity/weight control, dietary habits, and social inhibition, were found to be predictors of CAD prognosis. As several behavioral factors might be controlled through identification as subcategories of type-D personality, the combination of behavior-psychological factors should be separately identified and addressed to improve the prognosis of patients with CAD. Therefore, when preparing a rehabilitation program, combination-separating strategies for behavior-psychological factors are likely to be more effective in enhancing prognosis for CAD. 

## Figures and Tables

**Table 1:** Predictors of coronary artery disease (CAD) prognosis according to participant characteristics (*N* = 108).

Characteristics	Categories		N (%)	MetS Score	t/F(p)	Severity of CAD	Z/H(p)
Age (years)	40–49		13 (12.0)	3.15 ± 1.46	0.21	0.54 ± 1.66	9.66
	50–59		24 (22.2)	2.79 ± 1.53	(0.887)	1.13 ± 1.04	(0.022)
	60–69		39 (36.1)	2.95 ± 1.34		1.41 ± 0.99	
	70≤		32 (29.6)	2.88 ± 1.24		1.44 ± 0.98	
Gender	Male		56 (51.9)	2.93 ± 1.39	0.09	1.34 ± 0.98	−1.11
	Female		52 (48.1)	2.90 ± 1.33	(0.925)	1.15 ± 1.02	(0.269)
Educational	≤Elementary		26 (24.1)	2.77 ± 1.31	0.32	1.50 ± 1.07	0.02
Status	Middle		18 (16.7)	3.17 ± 1.51	(0.810)	1.44 ± 0.10	(0.880)
	High		54 (50.0)	2.89 ± 1.34		1.09 ± 0.96	
	≥University		10 (9.3)	3.00 ± 1.41		1.10 ± 0.74	
Marital status	Single		3 (2.8)	4.33 ± 0.58	1.86	1.00 ± 0.01	−0.32
	Married		105 (97.2)	2.88 ± 1.35	(0.066)	1.26 ± 1.01	(0.746)
Subjective	Low		19 (17.6)	3.47 ± 1.39	2.01	1.63 ± 1.12	4.14
Economic	Moderate		58 (53.7)	2.78 ± 1.26	(0.139)	1.28 ± 1.04	(0.126)
Status	High		31 (28.7)	2.84 ± 1.46		0.97 ± 0.75	
Subjective	Good		26 (24.1)	2.42 ± 1.47	5.79	0.96 ± 0.99	4.28
Health	Moderate		24 (22.2)	2.50 ± 1.18	(0.004)	1.13 ± 0.80	(0.118)
Status	Bad		58 (53.8)	3.31 ± 1.26		1.43 ± 1.05	
Smoking	Non-smoker		49 (45.4)	3.06 ± 1.35	0.51	1.29 ± 1.04	1.90
History	Past smoker		26 (24.1)	2.81 ± 1.30	(0.603)	1.04 ± 0.99	(0.386)
	Current smoker		33 (30.6)	2.79 ± 1.43		1.36 ± 0.93	
Family	Yes		90 (83.3)	2.94 ± 1.34	0.48	1.23 ± 0.96	−0.49
History	No		18 (16.7)	2.78 ± 1.44	(0.636)	1.33 ± 1.03	(0.625)
Type of CAD	Angina pectoris		22 (20.4)	2.86 ± 1.49	−0.21	0.68 ± 0.72	−2.99
	Acute coronary		86 (79.6)	2.93 ± 1.33	(0.838)	1.40 ± 1.01	(0.003)
	syndrome						
Antihypertensive		Yes	51 (47.2)	2.67 ± 1.37	−1.84	0.90 ± 0.83	−3.39
drug		No	57 (52.8)	3.14 ± 1.32	(0.69)	1.56 ± 1.04	(0.001)
Antidiabetic		Yes	79 (73.1)	2.62 ± 1.29	−4.01	1.20 ± 0.97	−0.72
drug		No	29 (26.9)	3.72 ± 1.19	(<0.001)	1.38 ± 1.08	(0.470)
Antihyperlipidemic		Yes	101 (93.5)	2.81 ± 1.32	−3.18	1.20 ± 0.98	−1.94
drug		No	7 (6.5)	4.43 ± 0.79	(0.002)	2.00 ± 1.00	0 (0.052)

MS = metabolic syndrome.

**Table 2:** Descriptive statistics of behavioral-psychological variables and predictors of CAD prognosis (*N* = 108).

Variables (Number of Items/Unit)	Mean ± SD	Actual Range	Potential Range
**Behavioral-psychological variables**			
Lifestyle (behavioral variables)			
Physical activity and weight control (8)	1.87 ± 0.74	1.00~3.50	1.00~4.00
Dietary habit (16)	2.32 ± 0.50	1.44~3.25	1.00~4.00
Drinking and smoking (3)	2.79 ± 1.01	1.00~3.67	1.00~4.00
Sleep and rest (3)	1.93 ± 0.50	0.67~2.67	1.00~4.00
Stress (2)	3.69 ± 0.90	2.00~4.00	1.00~4.00
Drug and health management (4)	2.30 ± 0.72	1.00~3.75	1.00~4.00
Type-D personality (psychological variables)			
Negative affectivity (7)	3.43 ± 0.82	0~4.00	0~4.00
Social inhibition (7)	2.71 ± 1.00	0~4.00	0~4.00
**Predictors for CAD prognosis**			
Metabolic syndrome score	2.92 ± 1.35	0~5.00	-
Triglyceride (mg/d L)	134.78 ± 71.17	38~428	-
HDL-cholesterol (mg/d L)	47.40 ± 14.38	28.0~94.6	-
Systolic blood pressure (mmHg)	131.75 ± 21.32	60~190	-
Diastolic blood pressure (mmHg)	78.80 ± 12.51	30~110	-
Glucose (mg/d L)	116.27 ± 39.27	69~244	-
BMI (kg/m^2^)	25.25 ± 2.90	18.2~32.0	-
Severity of coronary artery disease (%)	1.25 ± 0.99	0~3.00	0~3.00
Degree of left anterior descending artery stenosis	48.58 ± 36.83	0~100	0~100
Degree of left circumflex artery stenosis	29.62 ± 35.74	0~100	0~100
Degree of right coronary artery stenosis	35.05 ± 38.62	0~100	0~100

BMI = body mass index; HDL = high-density lipoprotein.

**Table 3:** Correlation between behavioral-psychological variables and predictors for CAD prognosis.

Variables	Lifestyle						Type-D Personality		MetS Score
	1	2	3	4	5	6	7	8	9
1. Physical activity and weight control	1								
2. Dietary habit	0.60 ***	1							
3. Drinking and smoking	0.29 **	0.52 ***	1						
4. Sleep and rest	0.44 ***	0.64 ***	0.50 ***	1					
5. Stress	0.66 ***	0.65 ***	0.45 ***	0.55 ***	1				
6. Drug and health management	0.08	0.37 ***	0.35 ***	0.33 ***	0.20 *	1			
7. Negative affectivity	−0.42 ***	−0.21 *	−0.06	−0.20 *	−0.30 **	0.10	1		
8. Social inhibition	−0.41 ***	−0.23 *	−0.13	−0.16	−0.25 *	0.04	0.76 ***	1	
9. MetS score	−0.22 *	−0.22 *	−0.10	0.08	−0.16	0.12	0.31 **	0.39 **	1
10. Severity of coronary artery disease	−0.51 ***	−0.53 ***	−0.25 **	−0.29**	−0.44 ***	−0.10	0.27 **	0.38 ***	0.21 *

* <0.05, ** <0.01, *** <0.001.

**Table 4:** Canonical correlation between behavioral-psychological variables and predictors of CAD prognosis.

Variables	Canonical Variate
**Set 1: Behavioral-psychological variables**	
Lifestyle (behavioral variables)	
Physical activity and weight control	−0.77
Dietary habit	−0.78
Drinking and smoking	−0.37
Sleep and rest	−0.40
Stress	−0.64
Drug and health management	−0.05
Type-D personality (psychological variables)	
Negative affectivity	0.52
Social inhibition	0.71
Percent of redundancies	15.14
**Set 2: Predictors for CAD prognosis**	
Metabolic syndrome score	0.59
Severity of coronary artery disease	0.91
Percent of redundancies	26.57
Canonical correlation	0.67
Significance test; F(p)	4.91(<0.001)
Variance explained	45.0%
